# Influential factors on urine EV DNA methylation detection and its diagnostic potential in prostate cancer

**DOI:** 10.3389/fgene.2024.1338468

**Published:** 2024-02-19

**Authors:** Ting Ding, Yanjun Diao, Xianfei Zeng, Lei Zhou, Guojun Wu, Jiayun Liu, Xiaoke Hao

**Affiliations:** ^1^ Fourth Military Medical University (Air Force Medical University), Xi’an, China; ^2^ School of Medicine, Northwest University, Xi’an, China; ^3^ Shanxi Lifegen Co., Ltd., Xi’an, China; ^4^ Department of Urology, Xi’an People’s Hospital(Xi’an Fourth Hospital), Xi’an, China

**Keywords:** extracellular vesicles, DNA methylation, prostate cancer, molecular diagnosis, urine biomarkers

## Abstract

The value of Extracellular vesicles (EVs) diagnostic markers is widely recognized. However, current research on EV DNA remains limited. This study investigates the biological properties, preprocessing factors, and diagnostic potential of EV DNA. We found that DNA positive vesicles account for 23.3% ± 6.7% of the urine total EV, with a large amount of DNA attached to the outside. EV DNA fragments are large, there is no significant effect on uEV DNA when store urine less than 6 h at 4°C. In addition, the influence of different EV extraction methods on methylation detection is also minor. More importantly, RASSF1A methylation in urine total EV DNA can distinguish between PCa and BPH, with an AUC of 0.874. Our results suggest the potential of urine EV DNA as a novel marker for PCa diagnosis. This provides a new idea for the study of urinary tumor markers.

## 1 Introduction

Liquid biopsy is a promising new technology for tumor detection, holding great potential owing to its capacity to address the challenges and heterogeneity associated with tissue biopsy sampling (Siravegna et al., 2017). Liquid biopsy targets three main components: circulating tumor cells (CTCs), circulating tumor DNA (ctDNA) and extracellular vesicles (EVs), each with distinct advantages (Pascual et al., 2022). Among these components, ctDNA is the easiest to obtain and is widely used for tumor diagnosis because of its numerous cancer-related molecular signatures. However, various problems persist in ctDNA detection. Firstly, this molecule, characterized by high fragmentation and a short half-life, primarily originates from apoptotic and/or necrotic cells. Then, due to the low percentage of ctDNA in total cell-free DNA (cfDNA), the detection of ctDNA can be prone to failure in the early stages of a tumor or with less sensitive detection methods, leading to false positives (Pascual et al., 2022). Moreover, clonal hematopoiesis (CH) mutations, resulting from the aging process of hematopoietic cells rather than tumor cells, contribute significantly to false positives in ctDNA detection (Heitzer et al., 2019; Pascual et al., 2022). Therefore, we hope to find a new liquid biopsy marker that can overcome the aforementioned challenges.

Although researchers have debated whether EVs carry DNA, the presence of DNA in EVs has been confirmed as research progressed (Qu et al., 2019; Elzanowska et al., 2021; Ghanam et al., 2022). Studies have shown that EV DNA primarily consists of dsDNA (Thakur et al., 2014), including internal and external DNA (Park et al., 2020). Unlike cell free DNA (cfDNA) derived from necrotic and apoptotic cells, EV DNA is actively secreted by living cells and more stable due to the larger fragment sizes and protection of vesicles (Garcia-Silva et al., 2020). Most importantly, EV DNA is highly consistent with the genomic DNA (gDNA) of the source cells (Thakur et al., 2014; Vagner et al., 2018), offering a theoretical basis for its use as a diagnostic marker.

Urine, as a truly noninvasive sample that can be readily obtained in substantial quantities, has been proven to be an excellent tool for the diagnosis of numerous diseases, especially urinary disorders (Zhu et al., 2021; Alahdal et al., 2023; Robinson et al., 2023). Many studies have confirmed the value of urine EVs in diagnosing various diseases (McKiernan et al., 2016; Connell et al., 2019; Barreiro et al., 2020). However, the biological properties of urine EV DNA, the influence of various storage and extraction methods, and its potential as a diagnostic marker remain unexplored. The objective of this study was to explore the biological properties of urine EV DNA, assess the influence of preprocessing, and tentatively evaluate its potential as a diagnostic marker.

## 2 Materials and methods

### 2.1 Patients

Twenty-six patients with PCa and eighteen with BPH that confirmed by pathology were recruited from the Department of Urology at the First Affiliated Hospital of the Air Force Medical University. Urine samples were collected with patient informed consent and approval from the hospital ethics committee (No.KY20222066-C-1). The patients were divided into two cohorts. The first cohort was used for analyzing the biological characteristics of EV DNA and factors influencing preprocessing, whereas the second cohort was used for the methylation detection of EV DNA under the preprocessing strategy established in the early stage. Clinical information for the patients is listed in Supplementary Material S1.

### 2.2 EVs isolation

EVs were isolated from freshly retained first-void urine samples (100 mL) using various methods. In the experiments concerning EV DNA biological characteristics, urine sample storage strategies, and urine EV DNA methylation diagnostic evaluation, we adopted the ultrafiltration combined with ultracentrifugation (UF + UC) extraction method as follows: urine samples were centrifuged at 300 *g* for 10 min and 2000 × g for 10 min, followed by ultrafiltration through a 100 kDa ultrafiltration membrane (Millipore, United States). After volume reduction to 1/10 of the original volume, the sample underwent centrifugation at 100,000 *g*× for 70 min. To investigate the potential impact of different EV extraction methods on EV DNA methylation detection, we introduced an alternative EVs extraction approach: ultrafiltration combined with precipitation (UF + precipitation) in the experiments assessing the influence of various extraction methods. In this method, urine samples were also concentrated through ultrafiltration (Millipore, United States), and then PEG precipitator (ExoQuick TC, SBI) was added and incubated at 4°C in a refrigerator for 2 h. EVs were obtained by centrifugation at 10, 000 × g for 60 min. The obtained EVs were re-suspended in PBS and were either used immediately in experiments or frozen at −80°C.

### 2.3 EVs characterization

The morphology of EVs was analyzed using transmission electron microscopy (TEM) (Tecnai, United States), as previously described (Maire et al., 2021). To examine the size distribution and particle concentration of EVs, fractionated samples were diluted in PBS for nanoparticle tracking analysis (NTA) using a ZetaView instrument (Particle Metrix, Germany). Western blotting (WB), following previous literature (Yu et al., 2021), was used to detect three EV-positive markers and one EV-negative marker: TSG101 (Abcam ab125011, 1:1,000), HSP70 (Abcam ab181606, 1:1,000), and CD9 (Abcam ab263019, 1:1,000). Calnexin was purchased from Proteintech (10,427-2, 1:500 dilution).

### 2.4 Nanoflow

To explore the proportion of DNA-positive vesicles in total EVs, a 100 μL aliquot of the EVs preparation, with a particle concentration of approximately 3×10^8^ particles/mL, was subjected (or not) to treatment with 0.2 U/μL RNase-free DNase I (Takara, 2270A) for 30 min at 37°C. SYTO 16 (ThermoFisher, S7578) in PBS was added to obtain a final dye concentration of 6 μM. The EVs samples were then incubated for 20 min at 37°C before nano-flow cytometry (nFCM) analysis.

### 2.5 DNA extraction and sulfite conversion

cfDNA was extracted from 10 mL of freshly retained first-void urine using the QIAamp MinElute ccfDNA Midi Kit (QIAGEN, Hilden, Germany) according to the manufacturer’s instructions. gDNA was extracted from the urinary sediment of 10 mL urine by QIAamp DNA Mini Kit (QIAGEN, Hilden, Germany) according to the manufacturer’s instructions. EVs were treated (or not) with 0.2-U/μl RNase-free DNase I (Takara, 2270A) for 30 min at 37°C, following which DNA was extracted also using the QIAamp DNA Mini Kit (QIAGEN, Hilden, Germany). DNA was measured using the High-Sensitivity dsDNA Qubit Assay (Invitrogen) and assessed using the Bioanalyzer DNA High Sensitivity Chip Kit (Agilent). DNA sulfite conversion was performed using the EpiTect Fast Bisulfite Conversion Kit (QIAGEN, Hilden, Germany).

### 2.6 RRBS

To explore whether different EV extraction methods have a significant effect on DNA methylation detection, genome-wide methylation analysis was performed using reduced-representation bisulfite sequencing (RRBS). Initially, the extracted DNA was digested with MspI and ligated to a methylated adapter with a complementary sticky end. The ligated product underwent bisulfite conversion and amplification to add Illumina sequencing indices. The libraries were subsequently quantified using the KAPA Library Quantification Kit for Illumina (KAPA, KK4844) and sequenced using an Illumina NovaSeq 6,000 sequencer in paired-end 150-bp mode. The NGS data generated by RRBS was initially processed using an in-house pipeline, starting from the FASTQ files to the aligned binary alignment/map (BAM) files. For each sample, the read adapters were trimmed using the trim_galore software (version 0.4.0), and the pair-end reads were merged into single fragments using the PEAR software (version 0.9.6). To eliminate synthetic CpGs, we eliminated 2 bp from each end of the merged reads. Subsequently, the processed reads were mapped to the CT and GA-converted human reference genome (hg19) using Bismark software (version 0.17.0) with Bowtie2 software (version 2.3.1), after which the methylation status of each CpG was called from the BAM files using Bismark. Samples with a bisulfite-conversion rate of <0.99 with 10x CpG sites below 500,000 were excluded from downstream analysis.

### 2.7 ddPCR

To preliminarily verify the diagnostic value of urine EV DNA, we selected RASSF1A, a gene widely recognized for its hypermethylation in PCa (Connell et al., 2019), and performed methylation analysis by Droplet digital PCR (ddPCR). This detection was performed using the Naica^®^ six-channel microdrop chip digital PCR system from Stilla technologies (France). PCR reactions were prepared with 10 ng sulfite-conversed DNA using the PerfeCTa Multiplex qPCR ToughMix (Quanta bio) within Sapphire chips (primers, probes, reaction system and amplification conditions listed in Supplementary Material S2). ddPCR was performed using a Naica Geode, programmed to partition the sample into droplets, followed by the thermal cycling procedure as suggested in the user’s manual. Images were acquired using a Naica Prism3 Reader and analyzed using Crystal Reader software for total droplet enumeration and droplet quality control, along with Crystal Miner software for extracted fluorescence values for each droplet.

### 2.8 Statistical analysis

In the experiments on the biological properties of EV DNA and its pre-processing effects, paired sample t-tests were employed to compare the two groups using GraphPad Prism (v9.4.1). Meanwhile, the unpaired *t*-test was used to analyze differences between groups when comparing methylation differences between PCa and BPH in different samples. Results are presented as mean ± SD, with statistical significance set at *p* < 0.05. Receiver operating characteristic (ROC) curve analysis was conducted using IBM SPSS Statistics (v27) to evaluate the diagnostic value of the RASSF1A methylation ratio in total EV DNA from urine. In addition, principal component analysis (PCA), pairwise correlation analysis and the unsupervised hierarchical clustering heat map drawing was conducted using R software (v3.3.2) to compare the methylation profiles of EV DNA obtained using different extraction methods.

## 3 Results

### 3.1 EVs extraction and identification

**FIGURE 1:**
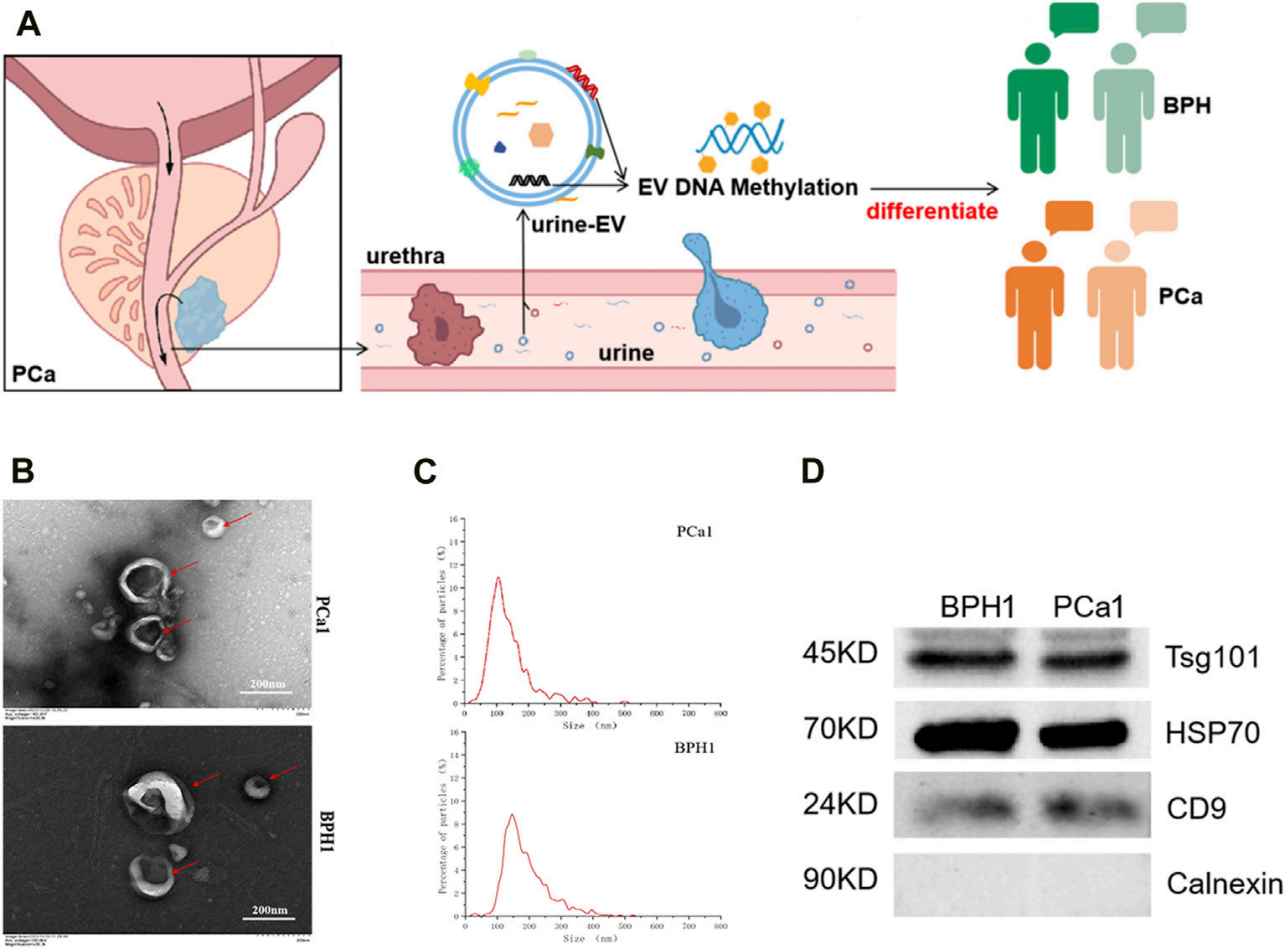
Extraction and identification of EVs. **(A)** Illustration of urine extracellular vesicles (u-EV) as PCa diagnosis marker. **(B)** Transmission electron microscopy images of EVs. **(C)** Nano particle tracking analysis of EVs. **(D)** Western blotting analysis of EV protein markers (TSG101, HSP70, CD9) and a negative control (Calnexin).

We characterized the extracted EVs from urine samples (Figure 1A) and selected the representative sample shown in the figure. The morphology was observed using transmission electron microscopy (TEM), revealing a typical cup-like shape (Figure 1B). Most EVs had a diameter ranging from 50 to 200 nm, which is clearly visible in the NTA (Figure 1C). We also tested for the presence of three “EV positive markers,” namely, TSG101, HSP70, CD9 and an “EV negative marker” Calnexin using WB (Figure 1D).

### 3.2 Biological characteristics of EV DNA

**FIGURE 2:**
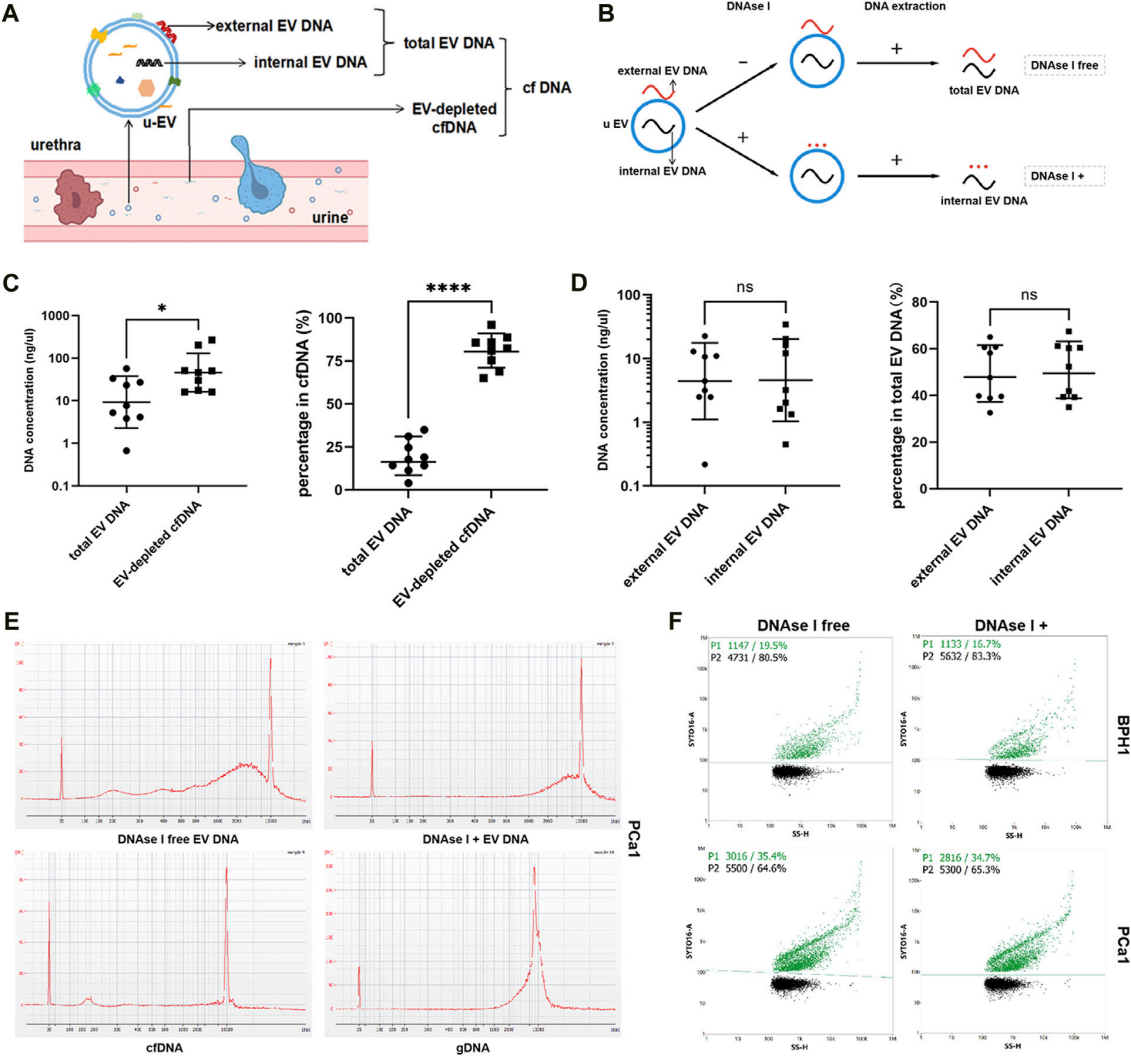
Biological characteristics of EV DNA. **(A)** Illustration of the different components of cfDNA in urine. **(B)** Illustration of EV DNA extraction. **(C)** Concentrations of total EV DNA and EV-depleted cfDNA and their proportion in cfDNA (EV-depleted cfDNA = cfDNA - total EV DNA). **(D)** Concentration of external EV DNA and internal EV DNA and their proportion in total EV DNA (external EV DNA = total EV DNA - internal EV DNA). **(E)** Capillary electrophoresis of EV DNA (with or without DNAse I), cfDNA and gDNA. **(F)** Nanoflow analysis of the proportion of DNA-positive vesicles in EVs treated with or without DNAse I.

The urine cfDNA actually contained both EV-depleted cfDNA and EV DNA fractions (Figure 2A). As shown in Figure 2B, EV DNA was extracted from urine samples obtained from nine patients (five with PCa and four with BPH) with or without the presence of DNase I. And cfDNA was extracted from 10 mL urine from the same patients. When comparing the concentrations of EV-depleted cfDNA and EV DNA in samples from the same patient, we first converted the cfDNA concentration to a concentration suitable for a 100 mL sample, then the concentration of EV-depleted cfDNA was then calculated by the concentration of cfDNA minus the concentration of EV DNA. In terms of content (concentration), EV DNA in urine accounted for 19.0% ± 9.8% of the total cfDNA in most samples (Figure 2C). In addition, some DNA was observed to adhere to the outer surface of EVs (Figure 2A). After DNase I treatment, the concentration of internal DNA accounted for 50.8% ± 12.1% of the total EV DNA (Figure 2D). Unlike cfDNA, which is highly fragmented, EV DNA displayed larger fragment sizes (Figure 2E, Supplementary Material S3). Subsequently, we used nanoflow to detect DNA-positive vesicles, and found that the proportion of DNA-positive vesicles in the total EVs was 23.3% ± 6.7%, with a slight change observed after digestion with DNAse I (Figure 2F, Supplementary Material S4).

### 3.3 Effects of different storage on EV DNA

**FIGURE 3:**
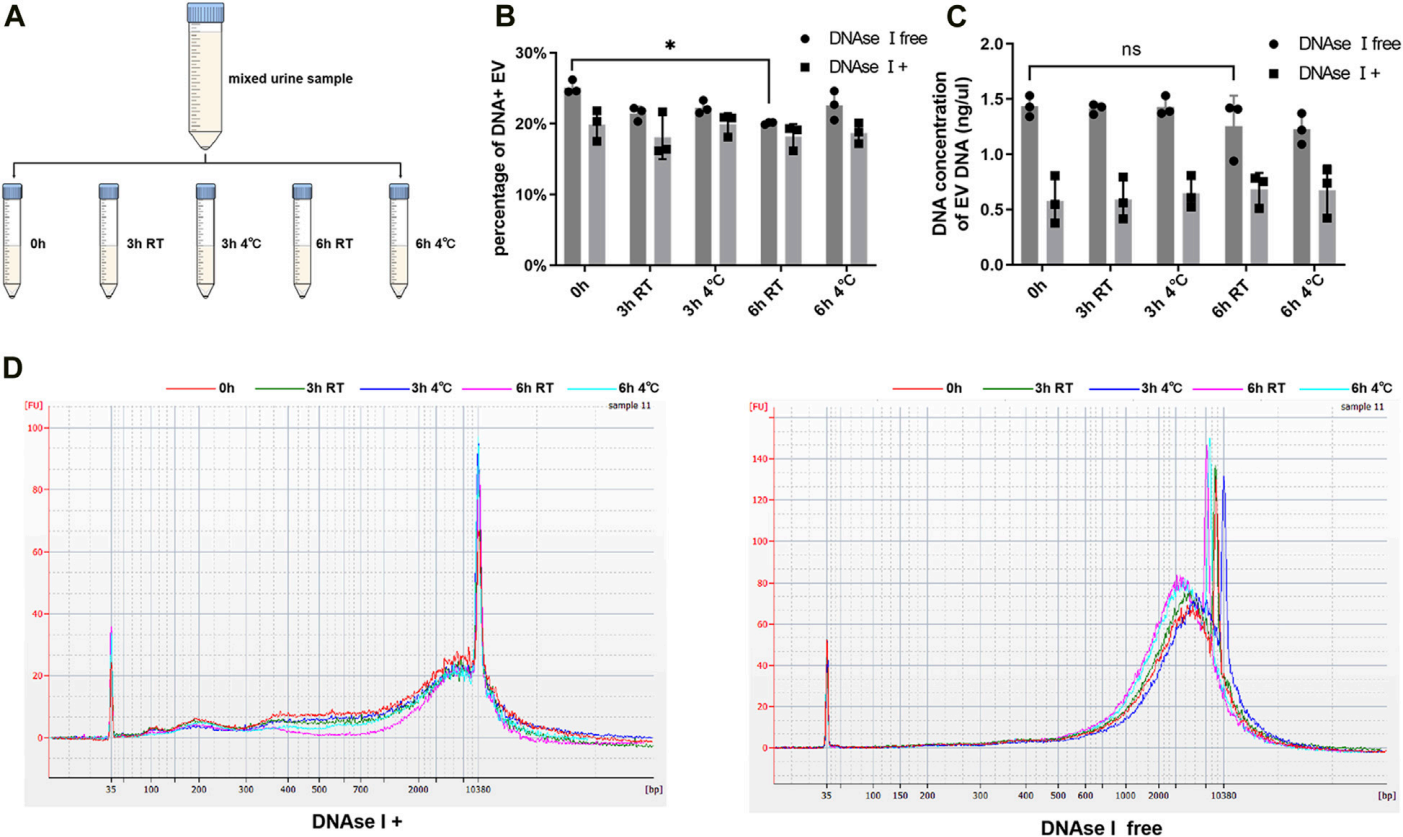
Effects of different storage on EV DNA. **(A)** Illustration of sample processing at different times and storage temperatures. **(B)** Nanoflow analysis of the proportion of DNA positive vesicles in EVs at different time and storage temperature (with or without DNAse I). **(C)** DNA concentration of EVs at different time and storage temperature (with or without DNAse I). **(D)** Capillary electrophoresis of EV DNA at different time and storage temperature (with or without DNAse I).

Urine samples of equal volume from nine patients (five PCa and four BPH) were combined and divided into five portions, with one part serving as the immediate control group, and the other parts were stored at either 4°C or room temperature (25°C) for 3 h and 6 h respectively (Figure 3A). This experiment was repeated three times. Subsequently, we assessed the DNA concentration and DNA fragment size of EV DNA before and after DNase I treatment. And we also compared the proportion of DNA-positive vesicles under different storage conditions. The proportion of DNA-positive vesicles significantly decreased after 6 h at room temperature without DNase I treatment (Figure 3B); however, the DNA concentration of EV DNA did not exhibit significant changes (Figure 3C). In addition, the results of capillary electrophoresis showed that the sizes of the DNA fragments of EV DNA in the different groups did not undergo significant alterations (Figure 3D). These findings suggest that the optimal approach is to store urine samples at 4°C after collection and process them within 6 h.

### 3.4 Effects of different extraction methods on EV DNA

**FIGURE 4:**
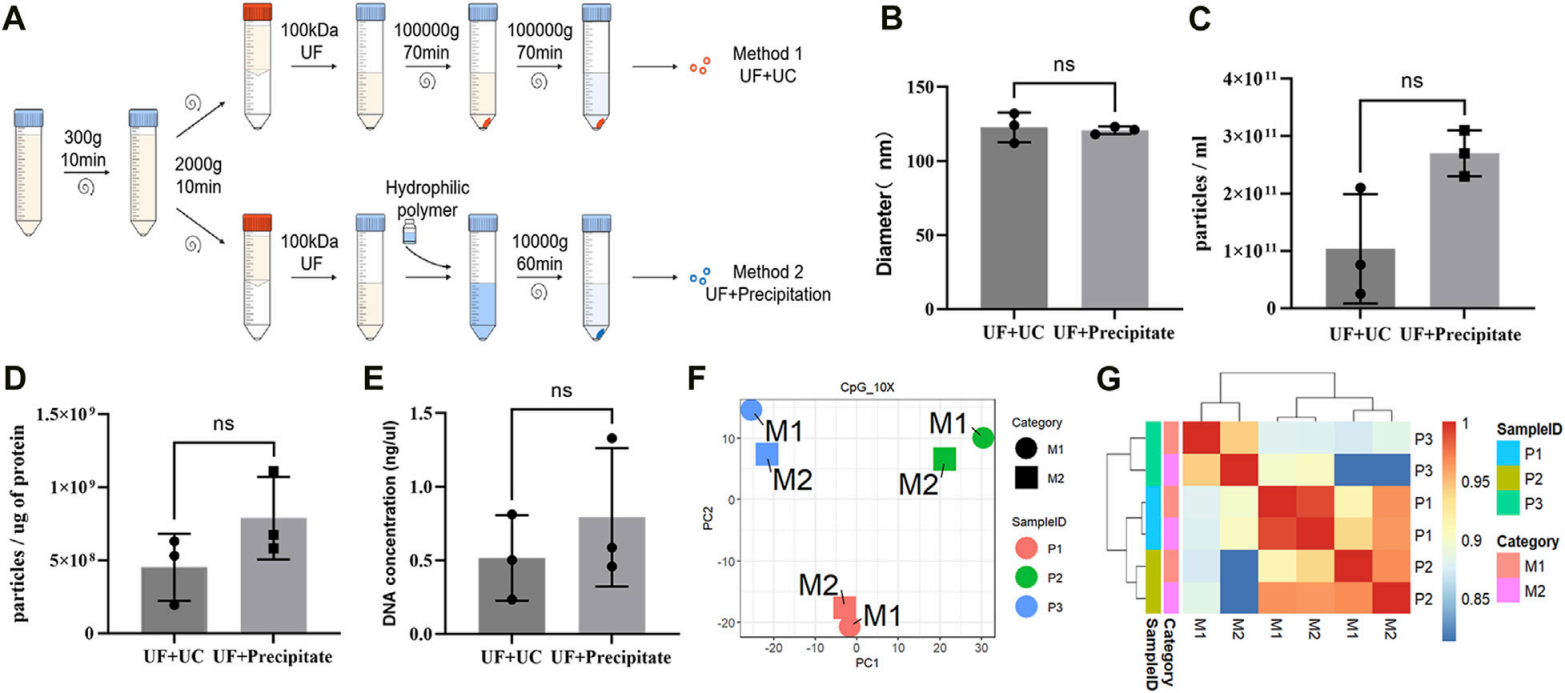
Effects of different extraction methods on EV DNA. **(A)** Illustration of different EVs extraction methods. **(B)** Mean particle size of EVs obtained by two extraction methods. **(C)** The number of EVs particles obtained by two extraction methods. **(D)** The ratio of particle to protein of EVs obtained by two extraction methods. **(E)** DNA concentration of EVs obtained by two extraction methods. **(F)** PCA analysis of DNA methylation profile of EVs obtained by two extraction methods. **(G)** Unsupervised hierarchical clustering heat map of the methylation profile of EVs obtained by two extraction methods.

To explore whether different EVs extraction methods had significant effects on DNA methylation detection, we used two different methods for the extraction of urine EVs from three patients (two with PCa and one with BPH). These methods included ultrafiltration combined with ultracentrifugation (UF + UC) and ultrafiltration combined with precipitation (UF + precipitation) (Figure 4A). Although the results were not statistically significant (Figures 4B–E), we found that, between the two methods, UF + precipitation yielded relatively higher values in terms of particle count (Figure 4C), purity (Figure 4D), and DNA concentration (Figure 4E). Subsequently, we conducted RRBS analysis on EVs obtained using different extraction methods from the same patient. PCA analysis and clustering analysis revealing that, the methylation profiles of samples from the same patient are very similar despite used different extraction methods (Figures 4F,G).

### 3.5 Urine EV DNA RASSF1A is hypermethylated in PCa patients

**FIGURE 5:**
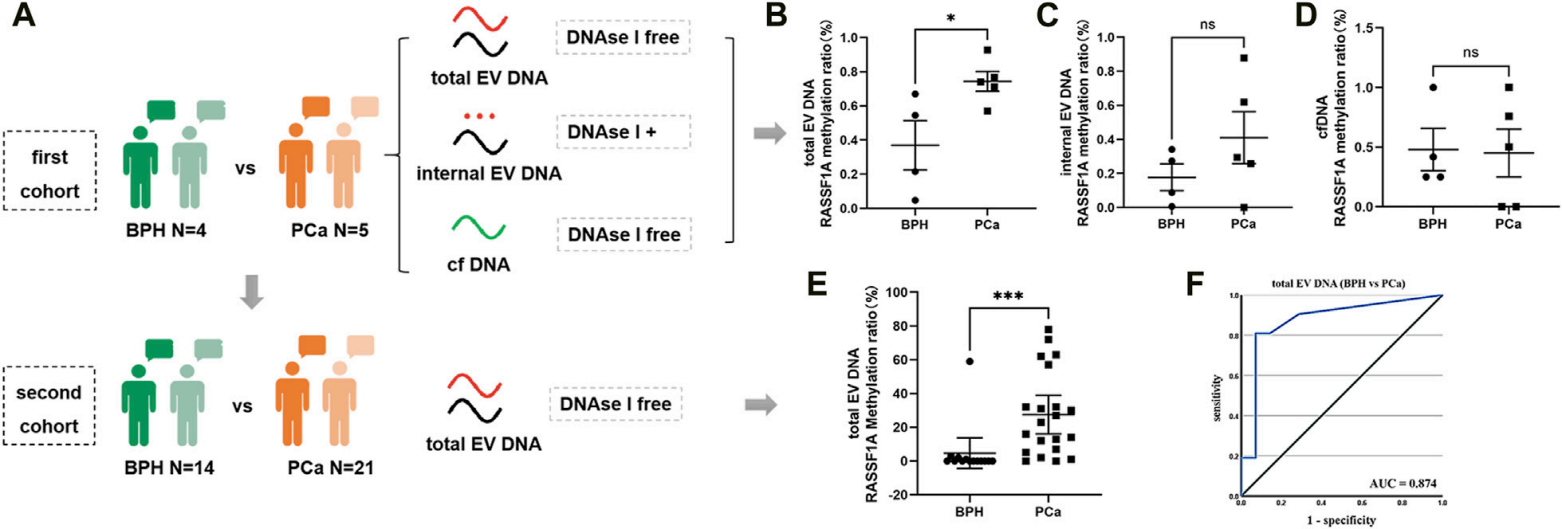
ddPCR detection of RASSF1A in urine. **(A)** Illustration of the different cohort detection processes. **(B)** ddPCR detection of RASSF1A methylation ratio in total EV DNA from urine on the first cohort. **(C)** ddPCR detection of RASSF1A methylation ratio in internal EV DNA from urine on the first cohort. **(D)** ddPCR detection of RASSF1A methylation ratio in urine cfDNA on the first cohort. **(E)** ddPCR detection of RASSF1A methylation ratio in total EV DNA from urine on the second cohort. **(F)** ROC curve of RASSF1A methylation ratio in total EV DNA from urine on the second cohort.

To preliminarily verify the diagnostic value of urine EV DNA, we selected RASSF1A, a gene widely recognized for its hypermethylation in PCa (Pan et al., 2013), and performed methylation analysis on urine samples from five PCa and four BPH patients, respectively (Figure 5A). Notably, we observed a significant disparity in RASSF1A methylation between PCa and BPH only within EV DNA without DNase I treatment (total EV DNA) (*p* = 0.03) (Figures 5B–D). This suggests that DNase I-free EV DNA samples may have higher sensitivity as potential PCa diagnostic markers. Furthermore, we quantified the methylation ratio of RASSF1A in total EV DNA samples from the second cohort (13 BPH vs 21 PCa). This analysis revealed a statistically significant difference between the two groups (Figure 5E, *p* = 0.0004), with an AUC value of 0.874 (Figure 5F). These findings underscore the potential of EV DNA as a promising tumor marker.

## 4 Discussion

PCa is one of the most prevalent malignancies in men, and its incidence is increasing in China and the rest of Asia due to aging populations and Westernized lifestyles (Zeng et al., 2018; Li et al., 2020). Although PSA is a cost-effective and easily detectable marker, the difficulty in distinguishing between benign lesions and tumors, especially in patients within the gray area of 4–10 ng/mL, may lead to unnecessary needle biopsies (Louie et al., 2015). Hence, there is an urgent need to identify more sensitive and specific markers.

EVs possess the unique ability to encapsulate the proteins, nucleic acids and metabolites of the source cells. Serving as the “cargo” carried by EVs, these components can serve as markers reflecting the state of the source cells. Moreover, they can facilitate intercellular communication, driving changes in the life activities of target cells (Cheng and Hill, 2022). While research on EV DNA components has been limited, it has garnered increasing attention in recent years (Macklin-Doherty, 2018). Similar to cfDNA, EV-DNA primarily comprises double-stranded and encompasses the whole genome (Louie et al., 2015). However, EV DNA offers several advantages over cfDNA. First, EVs are secreted by living cells and may provide an earlier and more accurate indication of tumor status compared to cfDNA from necrotic apoptotic cells. Second, unlike cfDNA, which cannot separate tumor-derived cfDNA, EV DNA can further improve detection sensitivity by enriching tumor-derived EV. Furthermore, EV DNA, characterized by larger fragment sizes, can provide more genetic information and demonstrates greater stablility (Qu et al., 2019; Elzanowska et al., 2021; Ghanam et al., 2022). Several studies have shown that EV DNA may exhibit greater consistency with tissue biopsy results and present advantages in early tumor diagnosis (Hur et al., 2018; Bernard et al., 2019; Park et al., 2020; Hagey et al., 2021). However, relevant studies on EV DNA in PCa are lacking; only the studies by Vagner et al. (Vagner et al., 2018) and Lazaro-Ibanez et al. (Lazaro-Ibanez et al., 2014) have tentatively explored EV DNA from the cell culture supernatants of PCa cell lines and plasma. Notably, the diagnostic potential of EV DNA in urine for PCa has not yet been reported. Given its anatomical proximity to the prostate and advantages for repetitive sampling and disease monitoring, urine, being a truly noninvasive sample, is ideal for PCa marker studies. Currently, some studies have explored the diagnostic value of urine EV in PCa, including the role of RNA (Diao et al., 2023; Jain et al., 2023), proteins (Wang et al., 2020; Bernardino et al., 2021) and metabolites (Clos-Garcia et al., 2018) in EV. Certain researchers have even developed diagnostic kits based on urine EV mRNA (McKiernan et al., 2016), indicating the feasibility of exploring PCa diagnostic markers in urine EVs. The unique advantage of EV DNA over other EV targets is its potential to contain genetic material from the tumor, which may offer distinct advantages in tumor genotyping and guiding treatment strategies. Therefore, we conducted this study to explore urine EV DNA as a diagnostic marker for PCa.

In this study, we explored the biological properties of EV DNA in urine. Our findings revealed that urine EV DNA fragments were larger than cfDNA fragments, which is consistent with previously published results (Qu et al., 2019; Liu et al., 2022). Additionally, we found that the proportion of EV DNA in the total cell-free DNA is usually <50% in urine, and the proportion of DNA-positive vesicles is approximately 30%, which has also been confirmed by other research groups (Liu et al., 2022; Sedej et al., 2022). Interestingly, upon treatment with DNAse I, the concentration of EV DNA was significantly reduced by approximately 50%, while the proportion of DNA-positive vesicles experienced only a minor change (Figure 2F, Supplementary Material S4). This suggests that most vesicles containing external DNA also harbor internal DNA. This implies that EV external DNA may involve more than simple adhesion to cfDNA, and that there may be selective mechanisms at play. However, this is speculative, and the origin of EV external DNA remains inconclusive. Further exploration is required to determine whether random adhesion of free DNA or selective assembly predominates, and under what conditions or disease states each mechanism is dominant.

Further, we examined the effects of various storage conditions on EV DNA and found that the fraction of DNA-positive vesicles decreased after 6 h at room temperature without DNase I treatment. In contrast, DNA concentration remained relatively stable. Interestingly, under the same storage conditions, the group treated with DNase I (preserving only DNA inside the EVs) did not show significant changes. This suggest that internal EV DNA is more stable, likely due to vesicle protection. The decrease in DNA-positive vesicles after 6 h at room temperature may result from minor degradation of external DNA or its release from the EVs, affecting the proportion of DNA-positive vesicles but having a limited impact on total DNA concentration.

Moreover, due to the relatively low EV DNA content, we referred to the EVs extraction method established by Xiaomei Yan’s team (Liu et al., 2022), with some optimizations, namely, UF + UC. Although this method has been used in many published studies (Merchant et al., 2017; Stam et al., 2021), it is time-consuming and inconvenient. As an alternative, we explored another extraction method and assessed its impact on the methylation profile. Our findings suggest that different extraction methods have only a minor influence on DNA methylation detection. In other words, clinical applications can choose more convenient EVs extraction methods without concerns about adversely affecting methylation detection.

Finally, we explored the feasibility of detecting urine EV DNA methylation as a diagnostic marker for PCa. Our results indicate that RASSF1A methylation is significant in PCa and BPH samples, but this significance is observed primarily in EV DNA samples without DNase I treatment. This indicates that total EV DNA may be more sensitive in reflecting genetic information from tumors, the external EV DNA may also carry a lot of information from the tumor. However, owing to the limited sample size, larger samples are required for validation to further illustrate the diagnostic value and advantages of urine EV DNA.

In summary, we determined the biological characteristics of urine EV DNA, explored urine storage methods for EV DNA extraction for the first time, analyzed the influence of different extraction methods on EV DNA methylation detection, and explored the potential of urine EV DNA methylation as a diagnostic marker for PCa. We believe that our findings contribute new insights to the study of tumor markers.

## Data Availability

The original contributions presented in the study are included in the article/Supplementary Material, further inquiries can be directed to the corresponding author.
