# Supplementary material for: Influential factors on urine EV DNA methylation detection and its diagnostic potential in prostate cancer
Source: Front Genet. 2024 Feb 19;15:1338468. doi: 10.3389/fgene.2024.1338468 (PMC10909848; doi:10.3389/fgene.2024.1338468)
Supplement: Supplementary file 1 [file DataSheet1.zip › Data Sheet 1/1. original image and datas/supplement 5 (ddPCR-cohort1).pdf]

BPH

sample	Nb Droplets	Blue Channel Dilution	Blue Channel C (cp/uL)	Blue Channel Nb Pos	Channel Dilution	Channel C (cp/uL)	Channel Nb Pos
BPH1-	22089	1	**0.85**	11	1	**3.09**	40
BPH1+	22456	1	**0.23**	3	1	**0.61**	8
BPH1cf	24721	1	**0.07**	1	1	**0.21**	3
BPH2-	22298	1	**0.31**	4	1	**6.29**	82
BPH2+	22591	1	**0.08**	1	1	**13.4**	177
BPH2cf	25301	1	**0.47**	7	1	**0**	0
BPH3-	21687	1	**0.79**	10	1	**0.39**	5
BPH3+	23315	1	**0.15**	2	1	**0.29**	4
BPH3cf	22630	1	**0.38**	5	1	**0.53**	7
BPH4-	24824	1	**3.79**	55	1	**3.17**	46
BPH4+	20277	1	**0.34**	4	1	**3.54**	42
BPH4cf	24875	1	**0.07**	1	1	**0.21**	3

- : DNAse I free ; + : DNAse I + ; cf : cfDNA

PCa

sample	Nb Droplets	Dilution	Blue Channel C (cp/uL)	Nb Pos	Dilution	Channel C (cp/uL)	Nb Pos
PCa1-	23682	1	**4.98**	69	1	**1.51**	21
PCa1+	25016	1	**0**	0	1	**0.48**	7
PCa1cf	23436	1	**0.15**	2	1	**0.15**	2
PCa2-	23941	1	**6.5**	91	1	**2.28**	32
PCa2+	25732	1	**0.73**	11	1	**1.39**	21
PCa2cf	18955	1	**0.09**	1	1	**0**	0
PCa3-	24342	1	**3.72**	53	1	**2.81**	40
PCa3+	22381	1	**1.14**	15	1	**0.61**	8
PCa3cf	23059	1	**0.3**	4	1	**0.07**	1
PCa4-	23446	1	**11.2**	153	1	**4.52**	62
PCa4+	25116	1	**1.7**	25	1	**2.92**	43
PCa4cf	23340	1	**0**	0	1	**0**	0
PCa5-	24884	1	**4.4**	64	1	**0.34**	5
PCa5+	24333	1	**3.23**	46	1	**0.35**	5
PCa5cf	23080	1	**0**	0	1	**0**	0

- : DNAse I free ; + : DNAse I + ; cf : cfDNA
