# Supplementary material for: Influential factors on urine EV DNA methylation detection and its diagnostic potential in prostate cancer
Source: Front Genet. 2024 Feb 19;15:1338468. doi: 10.3389/fgene.2024.1338468 (PMC10909848; doi:10.3389/fgene.2024.1338468)
Supplement: Supplementary file 1 [file DataSheet1.zip › Data Sheet 1/1. original image and datas/supplement 6 (ddPCR-cohort2).pdf]

BPH EV DNA

			Blue Channel			Channel	
sample	Nb Droplets	Dilution	C (cp/uL)	Nb Pos	Dilution	C (cp/uL)	Nb Pos
BPH1	27675	1	**0.06**	1	1	**2.22**	36
BPH2	17170	1	**0.1**	1	1	**16.3**	163
BPH3	28061	1	**0**	0	1	**18.5**	302
BPH4	26790	1	**0.19**	3	1	**66.7**	1027
BPH5	27379	1	**0.19**	3	1	**89.4**	1398
BPH6	18173	1	**0**	0	1	**19.6**	208
BPH7	17374	1	**0.1**	1	1	**5.51**	56
BPH8	28274	1	**0**	0	1	**5.56**	92
BPH9	27838	1	**0.06**	1	1	**26.1**	422
BPH10	23343	1	**0**	0	1	**23.8**	323
BPH11	23934	1	**0**	0	1	**17.8**	249
BPH12	24600	1	**1.18**	17	1	**0.83**	12
BPH13	18431	1	**0**	0	1	**4.17**	45
BPH14	27704	1	**0**	0	1	**2.16**	35

PCa EV DNA

sample	Nb Droplets	Dilution	Blue Channel C (cp/uL)	Nb Pos	Dilution	Channel C (cp/uL)	Nb Pos
PCa1	26654	1	**0.19**	3	1	**0.45**	7
PCa2	26958	1	**0.51**	8	1	**6.79**	107
PCa3	27403	1	**0.25**	4	1	**0.56**	9
PCa4	26377	1	**0.06**	1	1	**0.13**	2
PCa5	26512	1	**8.45**	131	1	**5.03**	78
PCa6	26388	1	**0**	0	1	**0.19**	3
PCa7	25853	1	**0.53**	8	1	**0.4**	6
PCa8	22683	1	**0.38**	5	1	**0.15**	2
PCa9	26045	1	**0.33**	5	1	**0.2**	3
PCa10	25480	1	**0.4**	6	1	**17**	252
PCa11	25952	1	**0.07**	1	1	**0.46**	7
PCa12	27369	1	**197.2**	2986	1	**54.7**	863
PCa13	27687	1	**0.86**	14	1	**4.63**	75
PCa14	26514	1	**1.03**	16	1	**3.54**	55
PCa15	27849	1	**0.12**	2	1	**0.92**	15
PCa16	26878	1	**0.51**	8	1	**1.08**	17
PCa17	25890	1	**0**	0	1	**0.79**	12
PCa18	27266	1	**0.13**	2	1	**2.69**	43
PCa19	25831	1	**8.15**	123	1	**21.8**	328
PCa20	26865	1	**0.06**	1	1	**4.2**	66
PCa21	27284	1	**0.06**	1	1	**0.38**	6
