# Supplementary figures and images for: Influential factors on urine EV DNA methylation detection and its diagnostic potential in prostate cancer
Source: Front Genet. 2024 Feb 19;15:1338468. doi: 10.3389/fgene.2024.1338468 (PMC10909848; doi:10.3389/fgene.2024.1338468)

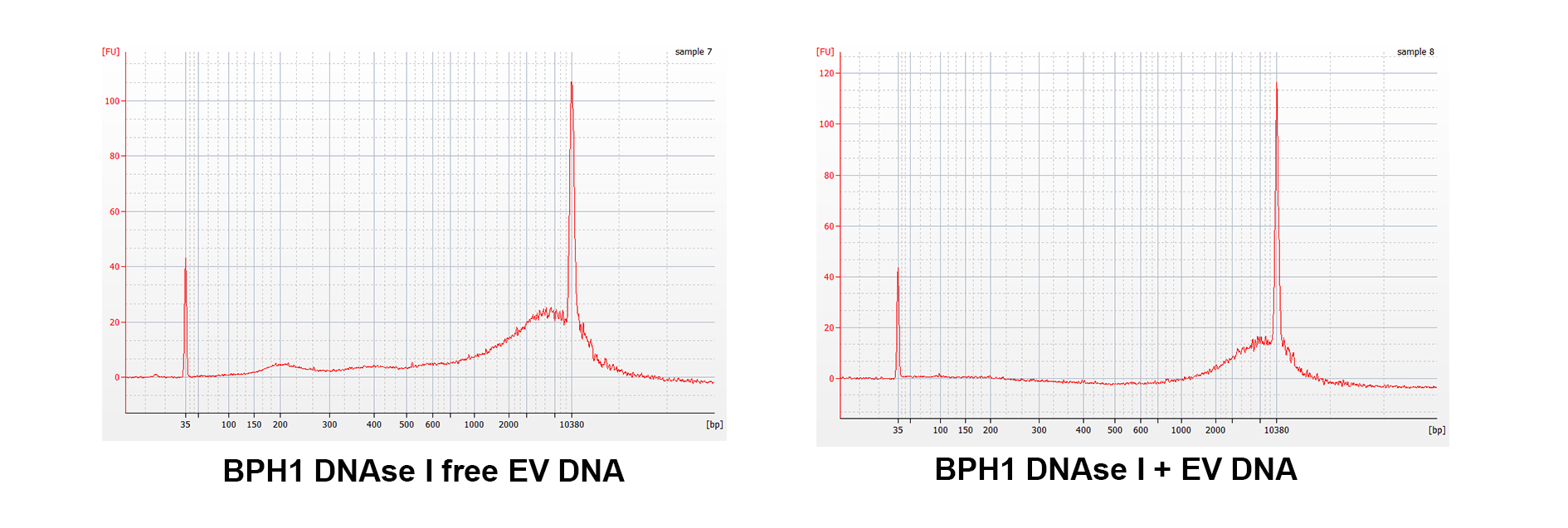

Supplement: Supplementary file 2 [file DataSheet2.ZIP › 2. supplementary material/supplement 3 (Capillary electrophoresis of BPH EV DNA).tif]

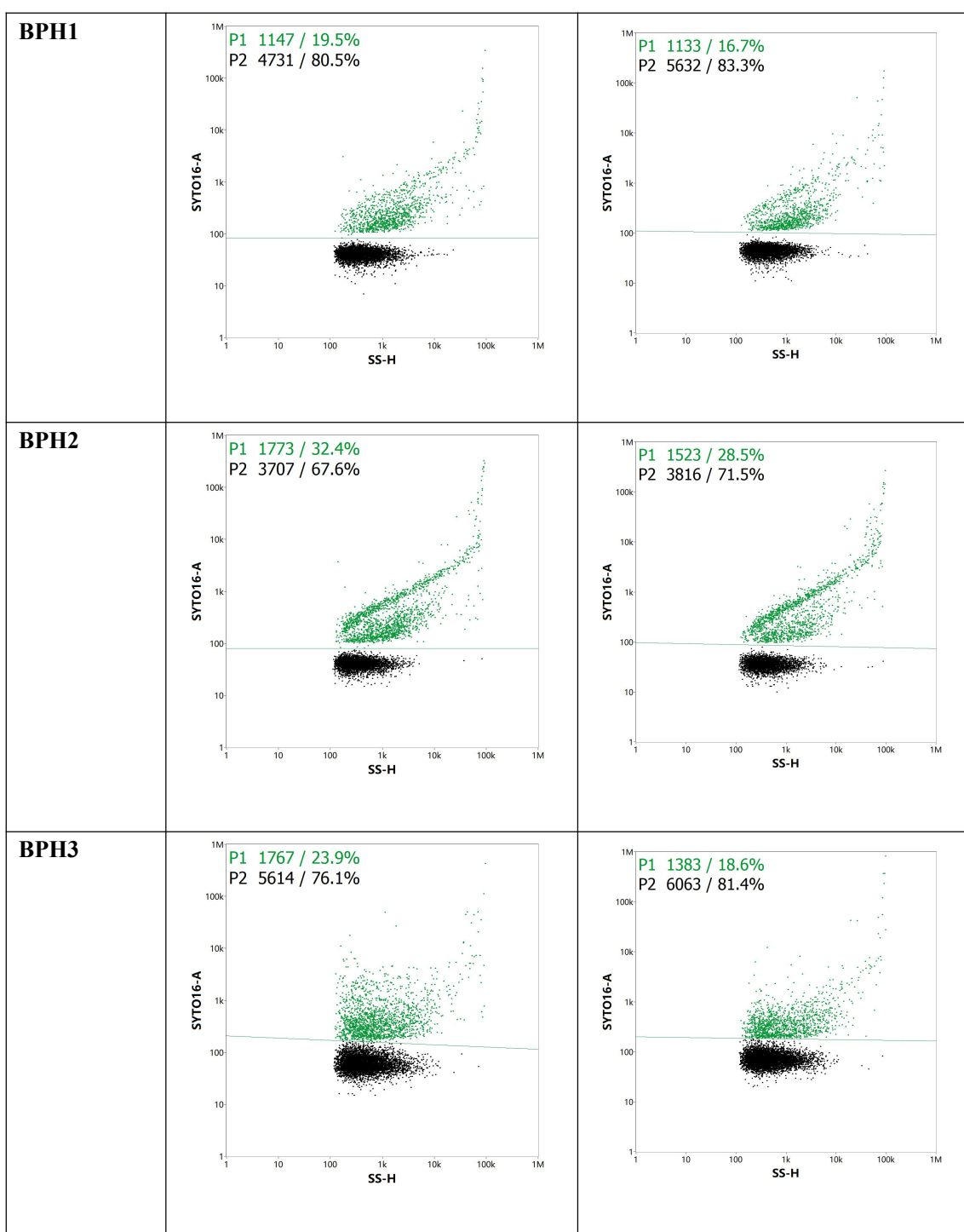

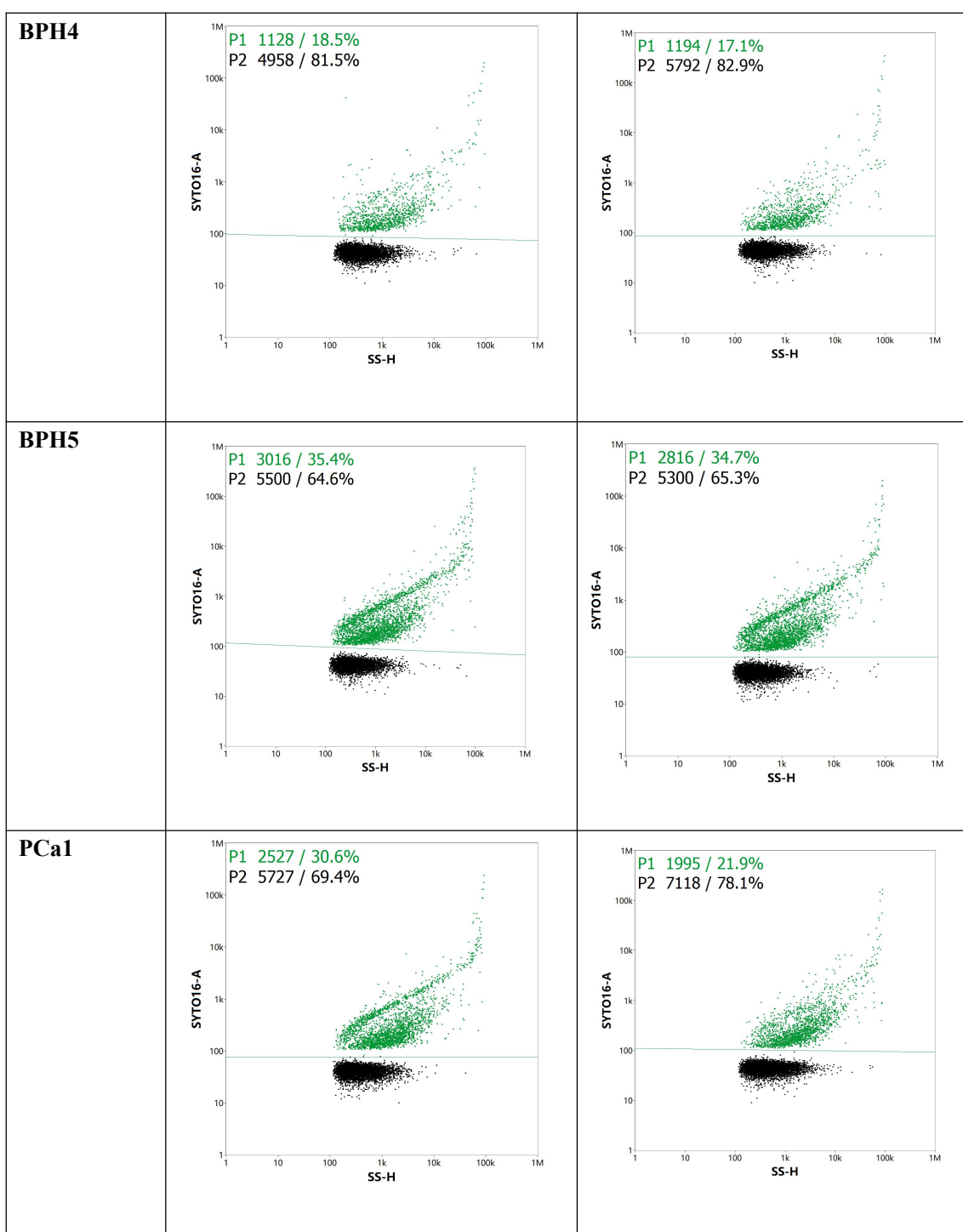

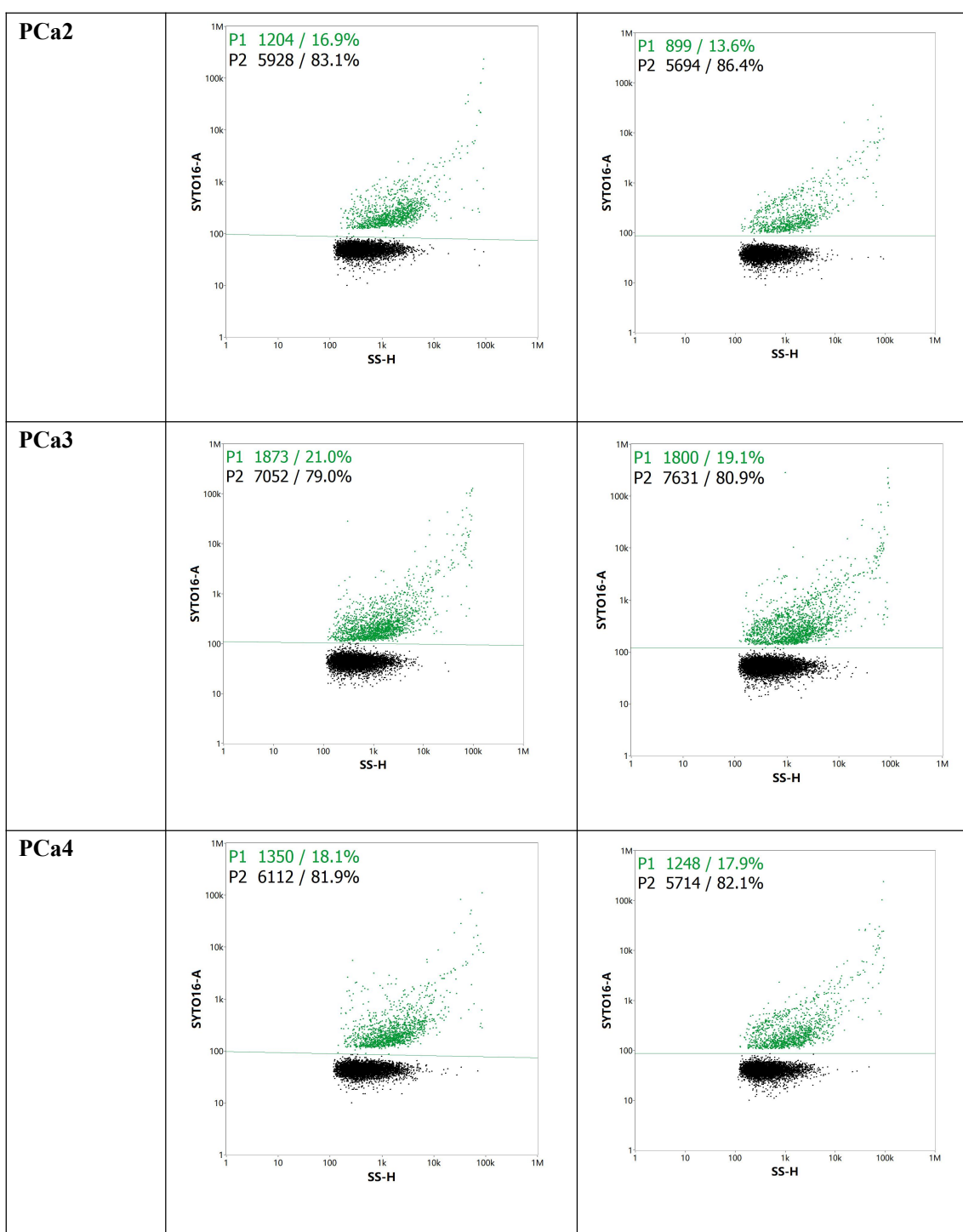

**PCa5**

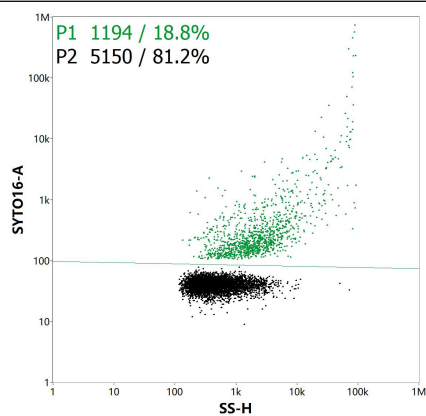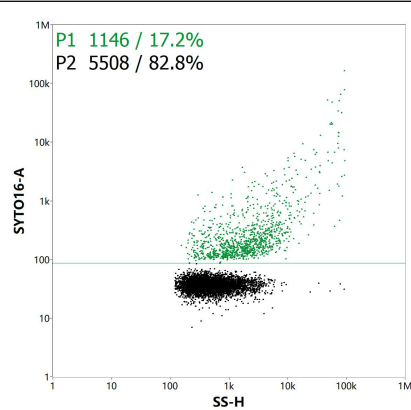

Supplement: Supplementary file 2 [file DataSheet2.ZIP › 2. supplementary material/supplement 4 (Nanoflow results).pdf]
